# Peer-to-Peer Sharing of Social Media Messages on Sexual Health in a School-Based Intervention: Opportunities and Challenges Identified in the STASH Feasibility Trial

**DOI:** 10.2196/20898

**Published:** 2021-02-16

**Authors:** Maija Hirvonen, Carrie Purcell, Lawrie Elliott, Julia V Bailey, Sharon Anne Simpson, Lisa McDaid, Laurence Moore, Kirstin Rebecca Mitchell, The STASH Study Team

**Affiliations:** 1 Department of Public Health and Policy London School of Hygiene and Tropical Medicine (see Acknowledgments) London United Kingdom; 2 MRC/CSO Social and Public Health Sciences Unit University of Glasgow Glasgow United Kingdom; 3 Department of Nursing and Community Health Glasgow Caledonian University Glasgow United Kingdom; 4 Department of Primary Care and Population Health University College London London United Kingdom; 5 see Acknowlegments

**Keywords:** social media, sexual health, sex education, peer education, process evaluation, school, feasibility studies, adolescent, social networking

## Abstract

**Background:**

There is a strong interest in the use of social media to spread positive sexual health messages through social networks of young people. However, research suggests that this potential may be limited by a reluctance to be visibly associated with sexual health content on the web or social media and by the lack of trust in the veracity of peer sources.

**Objective:**

The aim of this study was to investigate opportunities and challenges of using social media to facilitate peer-to-peer sharing of sexual health messages within the context of STASH (Sexually Transmitted Infections and Sexual Health), a secondary school-based and peer-led sexual health intervention.

**Methods:**

Following training, and as a part of their role, student-nominated peer supporters (aged 14-16 years) invited school friends to trainer-monitored, private Facebook groups. Peer supporters posted curated educational sex and relationship content within these groups. Data came from a feasibility study of the STASH intervention in 6 UK schools. To understand student experiences of the social media component, we used data from 11 semistructured paired and group interviews with peer supporters and their friends (collectively termed students; n=42, aged 14-16 years), a web-based postintervention questionnaire administered to peer supporters (n=88), and baseline and follow-up questionnaires administered to students in the intervention year group (n=680 and n=603, respectively). We carried out a thematic analysis of qualitative data and a descriptive analysis of quantitative data.

**Results:**

Message sharing by peer supporters was hindered by variable engagement with Facebook. The trainer-monitored and private Facebook groups were acceptable to student members (peer supporters and their friends) and reassuring to peer supporters but led to engagement that ran parallel to—rather than embedded in—their routine social media use. The offline context of a school-based intervention helped legitimate and augment Facebook posts; however, even where friends were receptive to STASH messages, they did not necessarily engage visibly on social media. Preferences for content design varied; however, humor, color, and text brevity were important. Preferences for social media versus offline message sharing varied.

**Conclusions:**

Invitation-only social media groups formed around peer supporters’ existing friendship networks hold potential for diffusing messages in peer-based sexual health interventions. Ideally, interactive opportunities should not be limited to single social media platforms and should run alongside offline conversations. There are tensions between offering young people autonomy to engage flexibly and authentically and the need for adult oversight of activities for information accuracy and safeguarding.

## Introduction

Young people (defined by the World Health Organization as 10-24 years) use social media as primary channels for digital interaction [1], and there is burgeoning interest in the potential of these media to convey sexual health information [2,3]. Relationships and sex may feature in young people’s exchanges on the web, and some see social media platforms as potentially important in supporting positive sexual health outcomes in their communities [4,5]. Nonetheless, in the context of everyday use, young people may not unreservedly accept sexual health content on social media [5-7]. There can also be a disconnect between health promotion conventions and the ways in which young people actually engage on social media [6,8]. To date, some interventions using social media have been shown to improve knowledge of sexually transmitted infection (STI) prevention and to potentially influence sexual behaviors; however, others have resulted in no effect [9-12].

Young people may have concerns about the reputational consequences of associating their social media profile with sexual—or other—health content; they may feel a need to carefully manage how they come across on social media platforms [4,6-8,13,14]. Attention to self-presentation and privacy are required to avoid rumors [7] and negative *drama*, including gossip and bullying [4,6]. Sexual health is potentially stigmatizing, and the avoidance of stigma is a strong factor shaping young people’s views [4-6,8]. A recent UK study of 16- to 19-year-olds suggested that the possibility of judgmental reactions from peers toward an overt link to sexual health content does dissuade young people from accessing such content [15].

Young people are aware of not only peers’ possible reactions but also those of parents, relatives, and other adults who may be *followers* [4,7,16]. They may consequently want to differentiate what specific social media contacts see and the difficulty of doing so—the concept of *context collapse* [17]—may further explain reluctance toward an association with sexual health content [8,16].

Certain factors can make it more acceptable for young people to access sexual health information or services using social media. These include receiving sexual health messages in an anonymous and unattributable manner and the use of youth-generated and humorous content [6,8,14], although the latter can sometimes miss the mark [14]. Young people also seek credible information from trusted sources [4,5,7,18]. They wish to participate in interventions in a way that allows them to control the degree to which they can be identified and for interventions to be monitored to prevent inappropriate behavior [4,5]. Formats such as question-and-answer forums or private messaging are appealing because of their potential anonymity and privacy [5,7,15,19].

Young people may talk about relationships, sex, and sexual health with each other both on social media and offline, although they may also question the trustworthiness, openness, and confidentiality of peer discussions and favor informal talks with close friends [4-6]. On social media, young people identify a role for friends in endorsing a sexual health intervention or service [5,7]. They may value peer role models in interventions [4,5], and peers’ real-life accounts—or fictionalized, plausible scenarios—as potentially effective intervention content [5,6]. Some young people wish to interact with each other as part of web-based, network-based sexual health promotion and knowing their contacts in real-life may be an important design criterion [5].

Although social media have become ubiquitous in health promotion [9-12], few interventions involve messaging instigated by young people themselves, and the knowledge of whether this would work in a school setting is lacking. This gap has held back innovation in school-based sexual health interventions, particularly with respect to informal and social norm–focused approaches that augment classroom learning. This study presents data from a feasibility study of an innovative intervention that employed social media in school-based, peer-led sexual health promotion [20]. We explored students’ views to draw out the opportunities and challenges related to the intervention’s use of social media and to contribute to wider debate about the use of social media in peer-led, youth-targeted sexual health promotion.

## Methods

### The Sexually Transmitted Infections and Sexual Health Intervention

The STASH (Sexually Transmitted infections and Sexual Health) intervention (Trial ID: ISRCTN97369178) was adapted from the effective peer-led antismoking ASSIST (A Stop Smoking in Schools Trial) intervention, premised on the diffusion of innovation theory [21,22]. Key adaptions for STASH were the focus on sexual health knowledge, beliefs, and behaviors (rather than on smoking); age group of participants (14-16 years in STASH; 12-13 years in ASSIST); and the use of social media to spread messages alongside offline conversations (ASSIST used conversations only) [20]. The aim was to reduce the risk of STI transmission and promote sexual health. The intervention recruited, trained, and supported students nominated by their friends to serve as peer supporters. During a 10-week intervention period, peer supporters were asked to share sexual health messages from the STASH website among their friends via Facebook (a private group function) and face-to-face conversations. Peer supporters also distributed the URL and password to access the STASH website (printed on cards) to their friends. The STASH website contains curated and bespoke relationships and sex education (RSE) content (memes, infographics, and links to other sites) and was co-designed with young people and health professionals as part of the intervention development.

Peer supporters invited friends to a private Facebook group in which they posted content from the STASH website (full details of the intervention and study is given in Forsyth et al [20]). The groups were monitored by STASH trainers (youth workers specializing in peer education), who were on hand to encourage the peer supporters, support with tricky questions, and guard against inappropriate posting. Peer supporters were also encouraged to chat to their friends about what they had learned in training and what they liked on the STASH website. The STASH intervention team opted to use Facebook because it was the only platform that offered the option of closed and monitored groups alongside direct sharing of content from the website (via a bespoke application programming interface) and log-in via the platform (without the need for personal information such as a telephone number).

STASH was implemented as a feasibility trial; full evaluation findings are reported elsewhere [23]. The intervention was delivered to 6 schools in central Scotland to assess the feasibility and acceptability of the approach, ahead of a full-scale evaluation. Here, we draw on process evaluation data sources that shed light on the role of social media within the overall intervention approach. The process evaluation sought to assess implementation, mechanisms of change, and context of the intervention via measurement of fidelity, acceptability, exposure, and reach [24]. In this paper, we focus on context, fidelity, and acceptability, specifically in relation to the social media component.

We use *peer supporters* and *friends* to distinguish between those who posted RSE messages (the former) and those who received messages (the latter) and *students* to refer to participating young people collectively.

### Setting and Participants

#### Paired and Group Interviews

We conducted 11 semistructured interviews with 42 students involved in STASH as part of the intervention’s process evaluation [20]; 6 paired or group interviews with peer supporters and 5 paired or group interviews with friends. The interviews were conducted with peer supporters (n=20; 9 young women) willing to participate on the day and friends they had invited to contribute (n=22; 10 young women; coordinated with the assistance of the STASH contact teacher). Of the 8 groups and 3 pairs of students, 6 were of single gender (3 all-young women and 3 all-young men) and 5 were of mixed gender. The interviews probed participants’ experiences and views of the STASH intervention. For the qualitative work, gender was based on self-identification at school (information on whether students were cis or trans was not collected).

We conducted interviews during the final 2 weeks of the intervention (December 2017). As a result of the constraints of the school setting and working within a single school period, interviews were relatively short (15-30 min). Participant information sheets were circulated to students and their parents or carers (along with opt-out consent forms), and opt-in consent was obtained from all interviewees on the day. All interviews were conducted by female researchers (including KM and CP).

Interviews were audio recorded and transcribed verbatim by a professional service. We carried out a thematic analysis of the qualitative data [25,26], using NVivo 11/12 software (QSR International) to facilitate data management. CP (lead for the process evaluation) coded the entire data set for the process evaluation and MH, using a separate coding frame, coded portions of the data set pertaining to the social media component for this paper. The analytic process for the process evaluation involved data familiarization, that is, summarizing and writing annotated memos; generation of a coding frame that married inductive observation with deductive attention to the study questions of interest; and review and refinement of codes (via discussion between KM, CP, and MH) [25,26]. Key themes were identified via synthesis of coding and discussion between analysts and with reference to the existing literature. The final themes presented here crystallized across the analysis stages and iterations of the write-up.

### Quantitative Data

To provide context to, and occasionally quantify, the qualitative themes, we included some descriptive statistics from quantitative data sources across the feasibility study: a web-based questionnaire completed by peer supporters at their final follow-up session (88/104, 84.6% response rate) and a student web-based baseline questionnaire (680/831, 81.8% response rate) and a follow-up questionnaire (603/744, 81.0% response rate, approximately 6 months later), administered to the intervention year group in all 6 schools. Facebook activity (counts of messages, likes, and replies) was extracted via the STASH trainer Facebook account with peer supporters’ consent (the trainer was a member of all groups). We also used Facebook Analytics reports (via an application programming interface between the STASH website and Facebook). Consistent with small sample size, exploratory analysis and minor scene-setting role, percentages are rounded to one decimal place, and confidence intervals are not calculated. Methods are not described in detail here, again because of their minor, scene-setting role. They can be found in detail elsewhere [20,23].

Ethical approval for the STASH study was granted by the University of Glasgow MVLS Ethics Committee (project number 20160002).

## Results

### Talking About the STASH Project and Sexual Health With a Researcher

Field notes taken during interviews record moments of silence and of youth whispering, mumbling, or giggling, and intermittently covering their faces. There was embarrassment when asked to recall message content. This discomfort was evident even among those who said sexual health content was not particularly unusual in social media exchanges; it was evident in those who had noted and read the messages and those who had ignored them.

### Variable Engagement With Facebook

Facebook monitoring data showed that across schools, an average of 83.6% (87/104) of peer supporters created a private Facebook group for STASH (8/19, 42% in one school in which Facebook was less commonly used and 79/85, 93% across the other 5 schools). The peer supporters invited an average of 12 friends (including on average 7 non-peer supporters friends) to their group, and they posted an average of 15 messages.

In the follow-up questionnaire, most students said they had a Facebook account (382/452, 84.5%), of whom 58.4% (218/373) said they looked at it regularly. However, 30% (21/70) of peer supporters reported to the peer supporter questionnaire that “the people I wanted to join my group hardly ever use Facebook.” This ranged from 0% in one school to 83% in another. It was reported more commonly among young men (27/70, 39%) than young women (17/70, 24%).

In interviews, not all the friends recalled the invitation to join a private Facebook group or the posts that were shared within them. Some friends said that they had not been added to the groups. Others described themselves as nonusers or infrequent users of Facebook or did not have notifications switched on. Taken together, these data suggested that using Facebook may have constrained the spread of messages across the school network.

Friends who did join found the groups acceptable; in the follow-up questionnaire, 60.2% (91/151) of students who were invited to STASH Facebook groups said they were happy to be a member and 35.0% (55/157) said they learned about sexual health by being part of the group.

### Private STASH Facebook Groups Were Reassuring but Ran Parallel to Routine Social Media Engagement

In the baseline survey, 68.0% (451/663) of students said that social media were “really important to [their] social life.” In interviews, students described using social media (eg, Snapchat and Instagram) routinely to connect with friends and, for some, that was the only purpose. They did not necessarily perceive a distinction between social media and offline interaction, with conversations threading through both modes of communication. Regular social media presence was common, and falling behind mattered, because “[*...*] then you don’t know the gossip the next [*...*] day” (friend, female), and it could mean that you “[*...*] miss out on a lot [*...*]” (friend, male). In the baseline survey, 15.8% (104/659) of students said they “often feel left out by what’s happening on social media” (68/364, 18.7% of young women; 35/282, 12.4% of young men).

Among peer supporters, a handful distinguished between their *own*, *actual*, or *real* Facebook use and their STASH group activity. Some said they would have reservations about posting STASH content openly on their personal social media, particularly where family members had been *friended*: “You’ve got your mum and dad and family on there. That’s weird” (peer supporter, female).

For many peer supporters, the fact that STASH involved posting messages to a private group whose membership they controlled, put them at ease with sharing sexual health content on social media:

...other people could, like, if you wanted to add them [friends from their year group] to the group or whatever, you could, like, use it, and then it was also, like, private, so, like, if you didn’t want anyone to see, they didn’t have to see it.

Peer supporter, female

Yeah. Like if there’s certain people you felt uncomfortable sharing that kind of information with, you didn’t have to add them.

Peer supporter, female 2

This separation of STASH activity from routine social media use was not universal: friends of peer supporters recounted how one peer supporter had promoted STASH on his or her personal profile (outside of the STASH private group), suggesting some variation in the ways that peer supporters combined STASH and routine activity.

Even with the private group option, 2 peer supporters spoke of initial disquiet around inviting friends to join:

It’s a bit awkward, adding folk, ‘cause you don’t know what they’re going to think of it.

Peer supporter, female

However, the apprehension about using Facebook seemed to largely dissipate, with others reflecting that, “it was actually alright” (peer supporter, female). The Facebook group format meant that friends could learn from posts without having to interact with anyone, and this was appreciated by friends: “[the group format meant that you weren’t] forced to message them if you know what I mean” (friend, female).

Having a trainer in the group did not appear to worry peer supporters or their friends. Indeed, 59% (42/71) of peer supporters said they were glad to have the trainer in their group (notably more young women than young men: 74% [31/42] vs 36% [10/28]; peer supporter questionnaire). In one group, some peer supporters felt that the trainer presence may have held back willingness to engage with the content and ask questions, whereas others suggested that students were more likely to be hindered by each other.

### The Offline STASH Context Legitimated and Augmented Web-Based Posts

Students in the intervention year group learned about STASH either via conversations with peer supporter triggered by the STASH training or via school bulletins and assemblies announcing the project. Some peer supporters recalled that their absence from school to attend training sparked curiosity among their friends and provided opportunities to discuss STASH. Comparison of baseline and follow-up questionnaire data suggested evidence of an increase in some STASH topics from pre to post intervention. For example, student reports of conversations with friends about STIs rose by 15.1% among peer supporters (from baseline of 26/96, 27%); 6.2% among students exposed to at least 1 STASH activity (from baseline of 35/237, 14.8%), and 1.8% among students who reported no exposure (from baseline of 45/214, 21.0%).

Friends of peer supporters who recalled seeing posts often described some initial surprise and subsequent contextualizing of the post as part of the STASH intervention, either by recalling previous conversations or via further discussions with peer supporters: “Yeah, once I saw [the Peer Supporters], they told us what [the invitation] was, obviously, and then that was it” (friend, male). Prior conversations tended to alert friends rather than inform them:

Interviewer: Did you – did you know what [the invitation to join the Facebook group] meant at that time?

Friend (male): I had an idea, but I didn’t know what it would be – [what] it’d be used for.

The personal connection mattered. The first post sent by peer supporters (which explained the purpose of the STASH groups) caught the attention of a friend in one interview, not because of the content itself but because it was a *serious* post sent by a peer supporter who typically shared humorous content.

The option in STASH to discuss content with message senders appeared to reduce both the unexpectedness and peculiarity of sexual health posts:

Interviewer: So then is it a bit weird then to suddenly get a sexual health education thing coming into [your routine social media activity]? Is that quite unusual?

Friend (female): Not really. They just put it in the chat and we talk about it.

Prior offline conversations also gave peer supporters confidence. In one group, a friend recalled conversations before one peer supporter first posted in her STASH Facebook group:

she was a bit wary of posting some things but we just kinda said, “Well you might as well, ‘cause it’s not like [other students are] gonna attack you for it. You were asked to so....

Friend, female

From the perspective of these friends, the fact of having been asked legitimated the sending of messages and protected the peer supporter from a negative reaction from peers.

Among peer supporters, the training also helped sensitize them to web-based sexual health content more generally:

[...] ‘cause I’ve been told about [sexual health] in an actual training session, I’d probably be more likely to read [a sexual health message] now. Be more interested.

Peer supporter, male

### Recipients of Messages Are Sometimes Receptive but Do Not Necessarily Engage Visibly on Social Media

Although some friends expressed disinterest in the STASH posts or looked at them out of boredom, others responded with openness and interest. One friend felt that the posts demanded attention because they were different from the usual content:

Friend (male): It was weird, ‘cause I don’t really – you don’t really see much o’ that (uhuh) posted on Facebook. (Uhuh). It was different to see.

Interviewer: So it was a bit different? Was it different good, or different bad, or different, just different?

Friend (male): It made you read it, ‘cause you don’t really see that.

Even when they were receptive to messages, friends rarely commented substantively on posts. According to the Facebook monitoring data, there were reactions (likes; comments; shares) to just over half of the posts. This was partly about not knowing how to respond or what to say, “[I] don’t know what to comment on that” (friend, female).

Similarly, although peer supporters were encouraged to modify the curated content or create their own messages, most peer supporters (50/88, 57%) said they preferred not to do this (peer supporter questionnaire) and only 2 individuals spoke of doing so in interviews. One described the need to alter a message to make it “more grown up” (peer supporter, female) and another to make a message stand out more:

...I felt as if someone was scrolling through their Facebook, it wouldn’t really stand out, people would just skip through it. So, I took a more creative approach and made it more interesting. Gave it a picture as well....

Peer supporter, male

The peer supporters were also proactive in overcoming technical issues, such as taking screenshots and sharing, when the application programming interface’s copy/share function failed.

### Preferences for Message Design and Content

Friends who were members of multiple groups were able to comment on the differences between them. During one interview, 2 friends noted that in one of their Facebook groups, content was conveyed using a humorous tone, but delivery was nonetheless direct, whereas in another group, messages were more discrete. Both were in mixed-gender Facebook groups, and they speculated that exchanges in single-gender groups may have been more open, though “[*...*] at the same time you want it to be mixed so you can see both sides” (friend, female). The instant notification feature of Facebook served to remind peer supporters to post regularly. It was also often seen as beneficial by friends; however, those in multiple Facebook groups sometimes viewed multiple notifications as annoying and subsequently muted them.

Participants commonly appreciated humor. They felt it helped draw attention and get messages across. Describing a meme of a nude cat, one participant said:

Yes, it was quite funny. It was something to make me read it...

Friend, male

Regarding web content in general, another said, “It made it funny but like*...*you got the information across” (friend, female). Bold colors were also viewed as attention grabbing:

...when you’re scrolling through and you just see like this big bright thing and you go, ‘Oh, what’s that’?

Friend, female

Brief, clear text and memes or pictures were viewed as “more interesting than reading like a chart or something like that*...*” (friend, male). Finally, balanced arguments were viewed positively:

And it was, like, giving you good and bad points, the ones that makes you, like, read more, that was more informative.

Friend, male

### Preferences for Social Media Versus Offline Interactions

Preferences regarding social media versus face-to-face communication varied among interviewees. One student explained that web-based interaction was less awkward than face-to-face, unless the chat was with a friend:

[...] when it’s with [...] one of your best friends it’s fine ‘cause it’s just funny.

Friend, female

In another interview, a student said that where others were unknown, web-based interaction was easier and less personal than face-to-face:

I don’t really mind it too much [being in a group with people you didn’t know], it’s not like – like actually, talking to them [...].

Friend, male

Other friends favored face-to-face conversation, seeing it as less antisocial (“*...*nobody’s talking ‘cause they’re on their phones’” [friend, female]), whereas others preferred a mix of social media and face-to-face interaction.

A couple of male friends said they would feel uncomfortable talking about sex on either channel but nonetheless recognized that social media posts could trigger discussion, especially when viewed by multiple members of the same friendship network:

It’s just ‘cause social media’s a thing that a lot of people use [...]...if it’s in a close group of people and if you all see the same post, you might end up like having a conversation, talking about it. If [...] the conversation comes up, and then it can start other questions, and then...So, it can be a good thing.

Friend, male

There were mixed views among peer supporters, with some seeing social media as “a good way to get across to other people in our year” (peer supporter, female) and others preferring to focus on offline conversations and awareness raising around the website. These mixed views were reflected in the peer supporter questionnaire, where 42% (36/86) said they preferred to talk, 30% (26/86) preferred to send messages, and 28% (24/86) were indifferent.

## Discussion

### Principal Findings

The STASH study was innovative in using student-led social media messaging alongside offline conversations in a peer-led, school-based sexual health intervention. We undertook a rigorous and theoretically informed process evaluation. In this study, we explored context, fidelity, and acceptability in relation to social media, to draw out opportunities and challenges of peer-led sharing of sexual health messages. At the time of implementing STASH, Facebook was the only platform to offer private groups, direct sharing of content from a website, and log-in via the platform (without the need for personal information such as a telephone number). However, student engagement with Facebook was variable and hindered message sharing where peer supporters, or their friends, were infrequent or nonusers. Invitation-only, monitored Facebook groups alleviated some peer supporter concerns around sharing sexual health content but led to engagement that ran parallel to their routine social media use. The offline context of STASH (trained peer supporters nominated by their friends; face-to-face conversations initiated by peer supporters) appeared to legitimate and augment web-based posts. Despite this and despite being generally receptive to the posts, few friends engaged visibly on social media. Preferences for content design varied; however, humor, color, and text brevity were important. STASH offered flexibility in ways of sharing messages, and there was no clear preference for either social media or offline message sharing among peer supporters and their friends.

### Comparison With Previous Work

Previous research has noted the role of social media in young people’s everyday communication with friends [4,6-8]; our data support these findings. Facebook use varied among STASH students, suggesting the need for alternative or multiple platforms. Facebook use among teens in the United States is declining and has recently been overtaken by YouTube, Instagram, and Snapchat [27]. The constantly evolving trends in young people’s engagement with social media, including the arrival of new platforms (eg, TikTok), underlines the importance of flexible engagement. Students’ design preferences confirmed the importance of offering a diversity of content types and allowing youth to shape messages (even if they rarely took this up in practice) [6,7]. Students actively shaped their engagement with STASH content (eg, opting out of notifications or screenshotting messages). This reinforces earlier conclusions on the importance of nuanced and flexible design, allowing prompt responses to changes in audience preferences and behavior [6,28,29].

The existing literature has discussed young people’s preoccupation with their image on social media and caution in associating with specific content in front of certain social media contacts, such as parents [5-8,16]. The notion of *context collapse* (audiences from disparate social contexts collapsed into one) has been invoked to help explain this phenomenon and the potential disruption caused by sexual health content to young people’s routine social media use [8,16]. In STASH, the private Facebook groups offered peer supporters a means to circumvent *context collapse* by encouraging them to invite only friends with whom they felt comfortable sharing sexual health content. Thus, as an artifact of this design feature, message sharing in STASH ran parallel to—rather than embedded in—routine use and interactions. The monitoring of Facebook groups by trainers, although acceptable to students, may also have suppressed the *usual* interaction. It likely prevented *drama* and bullying but also likely stifled positive visible engagement.

In line with earlier research [6-8], we detected signs of sexual health stigma, both in terms of sexual health as an interview topic and in the form of hesitation on the part of some peer supporters about distributing STASH messages publicly. Stigma may also have been a factor in the limited visible engagement of friends with STASH content in Facebook groups. Previous research has also indicated that some young people reject sexual health content on social media [6,7,15]. In this study, some peer supporter friends said they did not spend time viewing STASH messages or turned off notifications of new STASH posts. This may have reflected similar rejection on their part.

Notwithstanding the value placed on privacy and the stigma of sexual health, both our findings and the literature [4-8,14,15] suggest that employing social media in sexual health promotion does hold potential. Our results suggest that the offline context (trained influential peers supported by trainers) helped provide legitimacy to peer-led posting, that widely seen social media posts could trigger offline conversations among friends, and that social media could skirt the potential awkwardness of face-to-face conversations with peers. Yet, there was no universal preference for social media over face-to-face communication. Collectively, these findings lend further support to conclusions indicating that young people may not see social media as a viable sole component of effective sexual health interventions, emphasizing the contribution of offline resources and interaction [5,6,8,18].

### Strengths and Limitations

The strength of this study is that it draws from a rigorous and theory-driven process evaluation, which uses mixed methods to interrogate mechanisms of change within the intervention and examine feasibility and acceptability. We sought to achieve rigor via a detailed protocol [20], careful attention to possible reporting bias, and cautious and critical interpretation.

The necessity of conducting interviews within school periods imposed time limits that were shorter than ideal and limited the depth of discussion on social media. Field notes attested to a broader discomfort with discussion of sexual matters. This may have prevented students from admitting to a deeper interest in, or engagement with, the STASH messages. Such reluctance may have been amplified by the school setting and the awkwardness of talking about sexual health with an unknown adult in a position of authority. The paired and group interview format may also have influenced individuals’ inclination to discuss their views and experiences, particularly if they differed from others in the group.

### Recommendations and Conclusions

The STASH study demonstrates that peer-to-peer sharing of sexual health content via social media in school settings is feasible and acceptable. Our findings attest to the importance of multiple communication channels and opportunities beyond social media alone. They also suggest that to encourage buy-in, interventions must offer young people the flexibility and freedom to choose their preferred way of participation. In offering young people autonomy to engage in ways that are authentic to them, interventions must contend with possible tension brought on by adult oversight of activities for safeguarding purposes. To overcome concerns about association with sexual health content, young people may require a valid justification for message sharing; offline intervention activities can support this. In STASH, friends were often primed, received messages from a known and influential member of their social group, and had the opportunity to discuss content offline. This way, the broader intervention context both legitimated and augmented the social media component. Future studies could further explore which offline intervention activities are most effective at facilitating effective social media approaches.

Social media has strong potential; however, the challenges are significant. The fact that young people engage heavily with social media is, by itself, an insufficient justification to use it in sexual health promotion. It will only be effective if employed in ways that are authentic to young people, mindful of their priorities regarding web-based self-presentation and privacy, and credible as an information source [6,15].

## Abbreviations

ASSIST: A Stop Smoking in Schools Trial
RSE: relationships and sex education
STASH: Sexually Transmitted infections And Sexual Health
STI: sexually transmitted infection
